# Optimization of dual-saturation single bolus acquisition for quantitative cardiac perfusion and myocardial blood flow maps

**DOI:** 10.1186/s12968-015-0116-2

**Published:** 2015-02-19

**Authors:** Javier Sánchez-González, Rodrigo Fernandez-Jiménez, Nils D Nothnagel, Gonzalo López-Martín, Valentin Fuster, Borja Ibañez

**Affiliations:** Philips Healthcare Iberia, Maria de Portugal 1. 28050, Madrid, Spain; Centro Nacional de Investigaciones Cardiovasculares (CNIC), Melchor Fernandez Almagro 3. 28029, Madrid, Spain; Hospital Universitario Clínico San Carlos, Madrid, Spain; The Zena and Michael A. Wiener CVI, Mount Sinai School of Medicine, New York, NY USA

**Keywords:** Dual saturation acquisition strategy, Absolute quantitative cardiac perfusion, Cardiovascular magnetic resonance

## Abstract

**Background:**

In-vivo quantification of cardiac perfusion is of great research and clinical value. The dual-bolus strategy is universally used in clinical protocols but has known limitations. The dual-saturation acquisition strategy has been proposed as a more accurate alternative, but has not been validated across the wide range of perfusion rates encountered clinically. Dual-saturation acquisition also lacks a clinically-applicable procedure for optimizing parameter selection. Here we present a comprehensive validation study of dual-saturation strategy in vitro and in vivo.

**Methods:**

The impact of saturation time and profile ordering in acquisitions was systematically analyzed in a phantom consisting of 15 tubes containing different concentrations of contrast agent. In-vivo experiments in healthy pigs were conducted to evaluate the effect of R2* on the definition of the arterial input function (AIF) and to evaluate the relationship between R2* and R1 variations during first-pass of the contrast agent. Quantification by dual-saturation perfusion was compared with the reference-standard dual-bolus strategy in 11 pigs with different grades of myocardial perfusion.

**Results:**

Adequate flow estimation by the dual-saturation strategy is achieved with myocardial tissue saturation times around 100 ms (always <30 ms of AIF), with the lowest echo time, and following a signal model for contrast conversion that takes into account the residual R2* effect and profile ordering. There was a good correlation and agreement between myocardial perfusion quantitation by dual-saturation and dual-bolus techniques (R^2^ = 0.92, mean difference of 0.1 ml/min/g; myocardial perfusion ranges between 0.18 and 3.93 ml/min/g).

**Conclusions:**

The dual-saturation acquisition strategy produces accurate estimates of absolute myocardial perfusion in vivo. The procedure presented here can be applied with minimal interference in standard clinical procedures.

## Background

Myocardial perfusion is affected by several pathological situations, and the degree of myocardial hypo-perfusion correlates with clinical prognosis [1-3]. There is a need for safe noninvasive techniques that accurately quantify absolute myocardial perfusion. Cardiovascular magnetic resonance (CMR) has been proposed as a noninvasive radiation-free imaging tool for absolute quantification of myocardial perfusion, and its performance compares well with positron emission tomography (PET) [4].

Semi-quantitative methods based on the contrast enhancement ratio [5,6] or upslope index [7,8] have been proposed as a means of obtaining quantitative results from first-pass MR images; however, these approaches systematically underestimate myocardial perfusion reserve (MPR) compared with fully quantitative methods [9]. Fully quantitative measurement of absolute myocardial perfusion has been achieved with a dual-bolus protocol [10], in which a dilute bolus of contrast-agent is injected at the beginning of the acquisition, followed by a full-concentration bolus of the same volume [11]. The dilute bolus ensures a sub-saturating concentration of contrast agent in the left ventricle during measurement of contrast enhancement in the blood pool (arterial input function, AIF). The second bolus, of undiluted contrast agent, allows accurate estimation of contrast uptake by the myocardium. This method has been validated against microspheres in an animal model, showing a good correlation across low, normal, and hyperemic myocardial blood flow (MBF) [10], and a patient study showed good agreement with MPR estimates by PET [4]. However, clinical implementation of the dual-bolus method is not trivial because of the many manipulations required [11,12], limiting its inclusion in clinical routine. Moreover, the delay between bolus injections means that it is not possible to ensure acquisition of AIF and muscle information in the same cardiac situation.

An alternative approach is dual-saturation acquisition, in which AIF and myocardial tissue information are collected after injection of a single bolus of undiluted contrast agent [13]. In this approach, signal saturation during AIF definition is avoided by using a short saturation time, and myocardial tissue information is subsequently obtained with longer saturation times [13,14]. MBF estimation by this method requires careful control of three parameters: saturation times for accurate evaluation of contrast enhancement in the blood pool and cardiac muscle; R2* effects, especially during AIF estimation [15]; and the influence of k-space ordering during high resolution image read-out [16].

**Figure 1:**
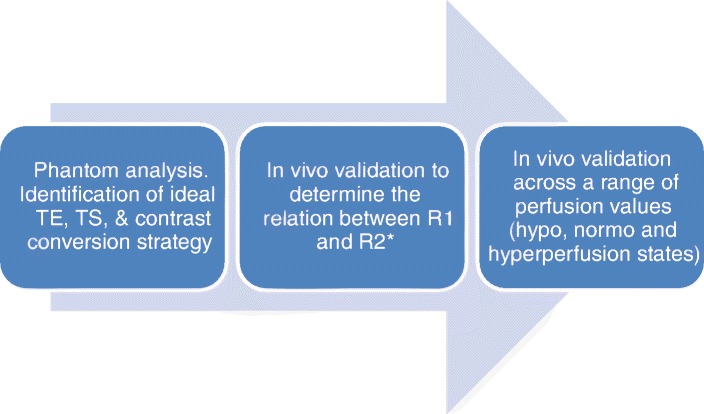
**Validation process.** Systematic validation followed in this work to assess accurate cardiac flow estimation by dual saturation technique.

The purpose of this study was to define a clinically useful procedure for accurate quantification of myocardial perfusion using the dual-saturation strategy. Using state-of-the-art 3Tesla-CMR, we analyzed the influence of saturation time (TS), echo time (TE), contrast injection rate, and image profile ordering on estimates of AIF and myocardial contrast uptake in a set of *in-vitro* (phantom) and *in-vivo* (large animal model) experiments (Figure 1). Signal acquisition and modeling derived from the in vitro analysis were incorporated in an in vivo comparison of the dual-bolus and dual-saturation strategies over a wide range of perfusion values, including hypo-perfusion (post-infarct), normal, and hyperemic MBF (pharmacological hyperemia), revealing generation of accurate MBF maps by dual-saturation CMR.

## Methods

All imaging experiments were performed on a 3T-TX Achieva platform (Philips Healthcare, The Netherlands) equipped with a 32-channel phased-array cardiac coil.

### Phantom experiments

The phantom consisted of 15 plastic 50 ml falcon tubes, each filled with distilled water and a specific concentration of contrast agent gadolinium diethylenetriamine pentaacetic acid (Gd-DTPA, Magnevist, Schering AG, Berlin, Germany), ranging from 0 to 22.5 mM. Three imaging experiments were performed with the same phantom to evaluate the effect of contrast concentration on R1 values and saturation times, and the influence of profile ordering on estimated final contrast concentration.

#### Estimation of R1 values at different contrast concentrations

Gold-standard T1 values of each tube were estimated using a look-locker inversion recovery acquisition with 147 inversion times ranging from 6.06-5886.06 ms, with a time interval of 40 ms. A new inversion pulse was applied every 10 s to avoid signal saturation. To reduce the influence of excitation readout at different inversion times, an excitation flip angle of 5° was applied during the TFE shot [17]. For tubes with R1 higher 20Hz, a second sequence was applied with lower TR and FA (TR/FA = 3 ms/2°). In both cases the final R1 values were estimated by fitting the signal intensity at different inversion times to the previously described signal model [17].

#### Evaluation of saturation times at different contrast concentrations

The second experiment was designed to evaluate the signal behavior at different saturation times and contrast concentrations. All parameters except TS were held constant (single shot TFE, TR/TE/FA = 2.26 ms/1.07 ms/15°), and ten images of each tube were acquired at TS = 10, 20, 30, 40, 50, 60, 70, 80, 90 and 200 ms. An additional proton density (S_PD_) image was acquired without saturation pulse to normalize the signal intensity of each contrast concentration (S/S_PD_). All images were acquired by low-high profile ordering to enable short TS and to avoid read-out effects on signal intensity.

#### Evaluation of the influence of profile ordering on estimated final contrast concentration

The third phantom experiment was designed to evaluate the influence of profile ordering on the conversion of MR signal changes into contrast concentration variations. This experiment was performed with 13 of the tubes with fixed TS of 100 ms. The read-out sequence was based on single-shot spoiled TFE acquisition (TR/TE/FA/TFEshot = 1.95 ms/0.9 ms/15°/55shots) with half-scan factors of 65% and linear and reverse-linear profile ordering. For each tube, we also computed signal intensity with the signal model described in the Appendix, using different T1 values and the same acquisition parameters in order to allow later comparison with real acquired values.

### Animal experiments

Experimental procedures were performed in castrated-male Large-White pigs. The study protocol was approved by the Institutional Animal Research Committee and conducted in accordance with recommendations of the Guide for the Care and Use of Laboratory Animals.

Pigs were sedated by intramuscular injection of ketamine (20 mg/kg), xylazine (2 mg/kg), and midazolam (0.5 mg/kg). A marginal vein in the ear was cannulated for peripheral intravenous access. Sedation was maintained by a continuous intravenous infusion of ketamine (2 mg/kg/h), xylazine (0.2 mg/kg/h) and midazolam (0.2 mg/kg/h).

The first *in-vivo* experiment was designed to evaluate the R1 and R2* variations at different contrast concentrations during first-pass perfusion, in order to establish the relationship between the two relaxation rates *in-vivo*. In a second *in-vivo* experiment, 11 pigs with different degrees of myocardial perfusion were examined simultaneously by the dual-bolus and dual-saturation methods to allow comparison of cardiac perfusion estimates by the two approaches. For hypoperfusion, 6 pigs underwent a closed-chest transmural myocardial infarction (45 minutes ischemia in the left anterior descending coronary artery followed by reperfusion); the procedure was performed with a dedicated percutaneous catheter inserted via the femoral artery [18,19]. Index CMR was performed 45 days post-infarction. For regional hyperemia, 4 pigs were subjected to intracoronary infusion with sodium nitroprusiate (4 mg/kg) or papaverine (0.5 mg/kg) during image acquisition inside the MRI scanner. Normoperfusion was assessed in 1 healthy animal undergoing CMR.

### *In-vivo* image acquisition

#### Evaluation of R1 and R2* changes at different contrast concentrations during first-pass perfusion

To define the relation between R1 and R2* values and contrast concentration *in-vivo*, different TS and TE were acquired during first pass of the contrast agent. To estimate R1 values every second, 6 saturation times were acquired over the range from 3.2-328.2 ms, with intervening gaps of 64.8 ms. The saturation times were acquired by sharing the same saturation pulse between 6 slice locations in transverse orientation along the descending aorta. The slices were acquired in ascending order to ensure that a saturated blood signal due to RF excitation pulses in one slice did not influence the signal acquired from the other slices. To estimate R2* values for every TS, four TE were acquired over the range from 0.8-3.8 ms with intervening gaps of 1.0 ms. The same experiment was performed dynamically every second over one minute, during which 0.1 mmol/kg of Gd-DTPA Magnevist (Schering AG, Berlin, Germany) was injected at a rate of 3 ml/s, followed by a 20 ml saline flush.

For these experiments, R2* values were obtained by fitting the signal at different TE to a mono-exponential model. The same procedure was applied for all TE values at different TS, and the mean was taken as the final result. R1 values were obtained by fitting the signal from different TS at the shortest TE to a regular saturation recovery model. After estimation of R2* and R1 for each dynamic acquisition, the values of the two quantities were fitted to a quadratic relation, as previously proposed [20].

#### Comparison between dual-bolus and dual-saturation methods

To evaluate the two quantification strategies, dual-saturation acquisition was combined with the dual-bolus injection protocol. For dual-bolus injection, the injection protocol was done using a single power MR injector Spectris Solaris (Medrad, Warrendale, Pennsylvania) using a previously described methodology by Ishida et al. [11]. Two boluses of Gd-DTPA Magnevist (Schering AG, Berlin, Germany) of equal volume and different concentration (0.01 mmol/kg and 0.1 mmol/kg) were injected at 3 ml/s, each followed by a 20-ml saline flush. A waiting time of 25 s was established between injections to ensure proper insulation of both curves during image analysis.

Three high resolution images (voxel size 2.6×2.6×10.0 mm^3^) and an additional interleaved low resolution image (6.8×2.6×10.0 mm^3^) were acquired at every R-R interval over 120 s. Interleaved low resolution image was acquired between the saturation pulse of the first high resolution image and image readout, sharing the same saturation pulse between low and high resolution images. To prevent any signal interaction between both images the planning was defined avoiding crosses between slices. When the heart rate was above 120 bpm one high resolution slice was removed from the acquisition to include all slices in a single RR interval. The image sequence consisted of spoiled TFE acquisition with a saturation time of 100 ms for high resolution images and 20 ms for low resolution images (TR/TE/FA = 2.0 ms/0.9 ms/15°). Images were acquired with reverse-linear profile ordering with a half scan factor of 75% and a SENSE factor of 2.6. All perfusion images were reconstructed to 2.0×2.0 mm^2^ in-plane resolution. To obtain baseline T1 values, an additional MOLLI (modified look-locker inversion recovery) T1 map was acquired with a 3–5 scheme [21] at the same location before every perfusion scan.

**Figure 2:**
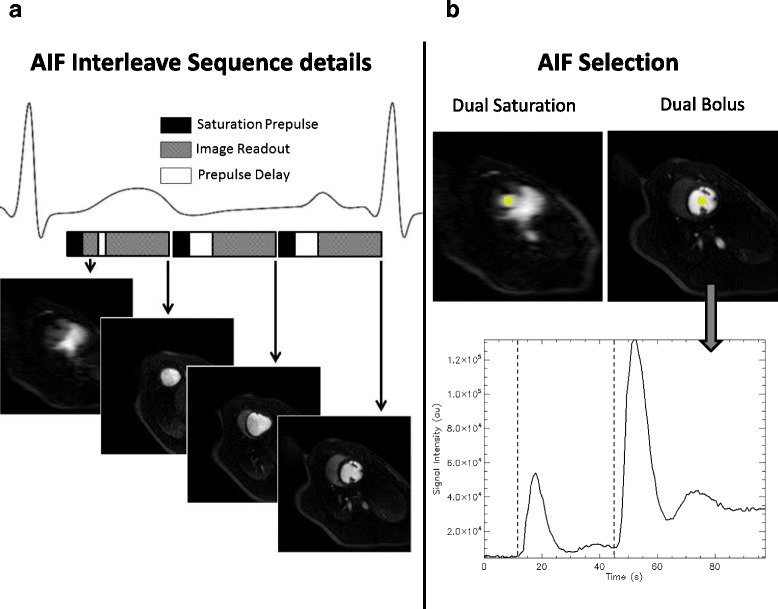
**Sequence Details and AIF drawing for Low and High resolution images. a)** represents the sequence details showing the short saturation time is interleaved acquired sharing the saturation pulse with the first high resolution image. **b)** ROI example for AIF selection in the dual saturation and dual bolus cases and an example of the signal-intensity curve at the high resolution. Dotted vertical lines represent the selection of the limits for diluted AIF.

The full image analysis to obtain flow information was performed using custom software written in IDL8.1 (Exelis Visual Information Solutions, Boulder, Colorado). Time-intensity curves were transformed into concentration curves using the methodology described in the Appendix for low and high resolution images and both perfusion strategies. In dual-bolus analysis AIF was defined by drawing a region of interest (ROI) in the left ventricle cavity of high resolution images and manually choosing the region of the time curve corresponding to the first diluted contrast as the portion of the curve between the first and second upslope shoulders of the intensity curve (Figure 2b). The duration of the final AIF was established as the time difference between both temporal positions and the initial point was established at the upslope shoulder of the full concentration injection (dashed lines in Figure 2b). After signal cropping the diluted AIF was temporary registered to the full contrast AIF to prevent shift with the muscle contrast uptake by automatic method that find the maximum correlation between both curves by temporal shifting of diluted AIF. Finally, the diluted, cropped and registered AIF was scaled according to the concentration difference. In dual-saturation analysis an ROI was drawn on low resolution images with no scaling factor and using the whole time curve in the deconvolution process. Model-based deconvolution was used to estimate flow, using an exponential model with 2 fitting parameters:

5 $$ \mathrm{h}\left(\mathrm{t}\right)=\mathrm{F} \exp \left[-\mathrm{k}\mathrm{t}\right], $$

where F represents the magnitude of the function, directly related to the blood flow, and k describes the decay rate of h(t) due to contrast wash-out. No shift was included in the model since AIF and cardiac muscle contrast uptake were measured close enough, making such correction unnecessary. Cardiac flow was assessed by scaling the F value according to a muscle density of 1.05 g/ml and assuming a constant hematocrit of 0.35 following high permeability model

6 $$ MBF=\frac{F\left[mi{n}^{-1}\right]}{\left(1- Hematocrit\right)*1.05\left[g/ ml\right]} $$

After flow-map generation, a quantitative comparison of the dual-bolus and dual-saturation methods was performed by ROI analysis in the ischemic or hyperemic region and the contralateral wall for all acquired slices and animals. The ROI was defined in the dual-bolus flow maps and was copied to dual-saturation maps to extract the final numerical data. Correlation and Bland-Altman plot analysis were performed to compare flow measured with the two approaches.

## Results

### Phantom experiments

#### Estimation of T1 values at different contrast concentrations

Table 1 shows the T1 and R1values of the different contrast concentration tubes. These values were taken as the reference T1 values to assess signal behavior at different contrast concentrations in subsequent phantom experiments. The T1 values ranged from 3693 ms for the phantom without contrast agent to 14 ms for a contrast concentration of 22 mM. This contrast concentration corresponds to the upper limit observed in human experiments with a 0.1 mmol/kg contrast injection at a rate of 6 ml/s [22].

**Table 1:** T1 and R1 values of the phantom tubes

**T1(ms)**	**R1(Hz)**
3693	0.27
2685	0.37
641	1.56
581	1.72
451	2.22
400	2.50
335	2.98
304	3.29
255	3.92
208	4.80
138	7.22
70	14.31
34	29.01
21	48.71
14	71.69

#### Evaluation of saturation times at different contrast concentrations

**Figure 3:**
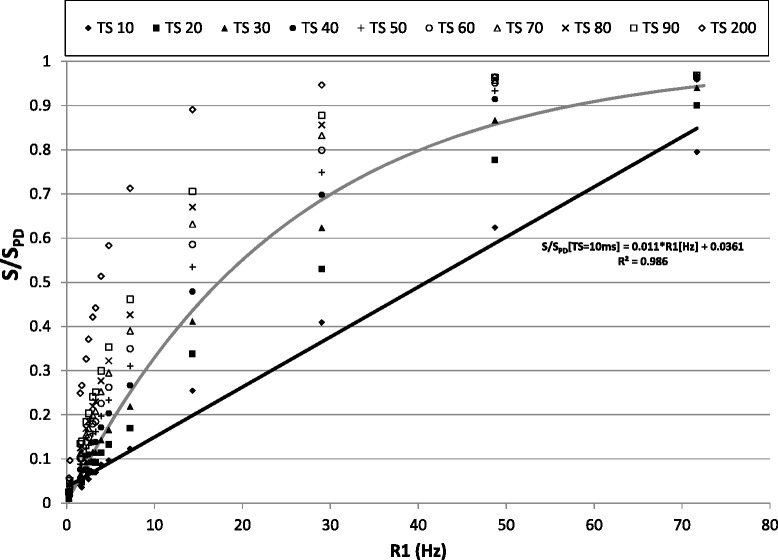
**Signal intensity for different Saturation times at several contrast concentration.** Signal acquired for different contrast concentrations at different saturation times (10, 20, 30, 40, 50, 60, 70, 80, 90 and 200 ms). The intensity values for each tube (S) were normalized to the signal intensity of the image acquired without saturation pulse, which represents the fully recovered signal after saturation (S_PD_). The solid black line represents the linear fitting of S/S_PD_ for each tube and different R1 values. The solid gray line represents the conventional saturation recovery signal model for a saturation time of 40 ms.

The effect of the TS on signal intensity at different contrast concentrations is shown in Figure 3. For a TS of 10 ms, signal (S/S_PD_) increased linearly with contrast concentration in the phantom, with a linear fitting correlation coefficient of R2 = 0.986. In contrast, for a TS above 40 ms the signal was fully recovered at higher contrast concentrations, with a value of S/S_PD_ close to 1, saturating the signal response.

#### Evaluation of profile ordering in estimates of final contrast concentration

**Figure 4:**
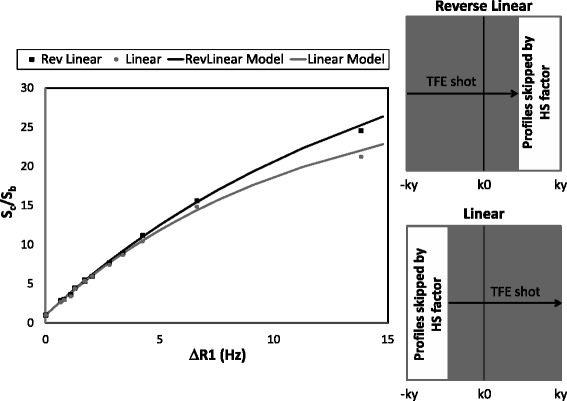
**Read-out effects on signal intensity.** The graph on the left represents the signal ratio between the signal of the phantoms at different contrast concentrations and the signal of the tube without contrast for Reverse Linear (black squares) and Linear (gray circles) profile ordering. Dotted lines represent the result of the same ratio derived from the signal model described in the appendix for both profile ordering schemes. Solid lines represent the result of the signal model of S_c_/S_b_ described in the Appendix for both readout strategies.

The effect of different readout strategies on the signal intensity ratio at different contrast concentrations is represented in Figure 4. Signal intensity ratios were calculated by dividing the measured signal intensity of each tube by the measured signal of the tube filled with pure water, as described in the Appendix. Linear correlation analysis between the measured S_c_/S_b_ data (points in Figure 4) and the signal model (solid lines) yielded correlation coefficients of R^2^ = 0.999 and R^2^ = 0.998 for reverse-linear and linear profile ordering, respectively. The slopes between the model and the measured data were 0.979 and 0.978 for reverse-linear and linear profile ordering, respectively.

### In vivo data

#### Evaluation of R1 and R2* changes at different contrast concentrations during first-pass perfusion

**Figure 5:**
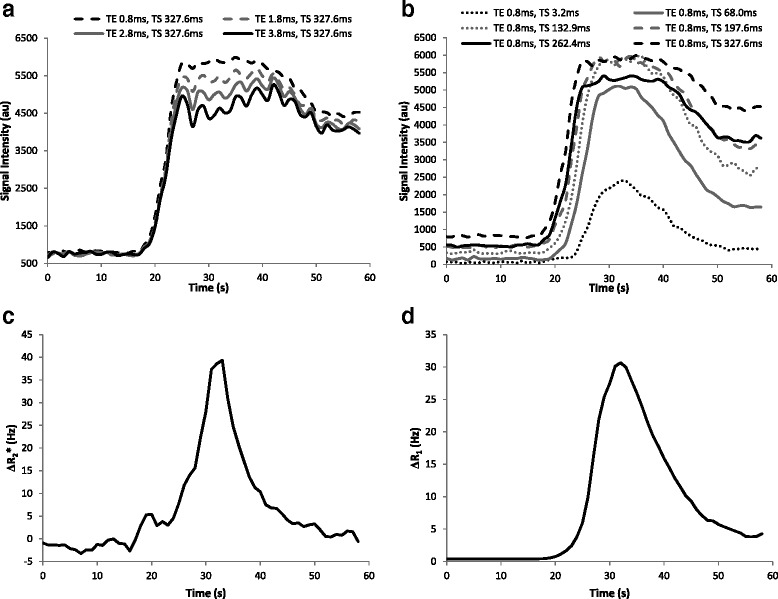
**In-vivo effect on the MR signal due to single bolus contrast injection.** Example of the signal variation in the aortic blood for different contrast concentrations and different echo times **(a)** and different saturation times **(b)**, and the corresponding results of the R2* **(c)** and R1 **(d)** variations.

MR signal changes due to R1 and R2* effects during the first-pass of bolus injection are shown in Figure 5. For longer TE (Figure 5a) there is a drop in signal due to R2* effects at very high contrast concentration. The effect of R2* changes (∆R2*) reached a maximum value of 39Hz (Figure 5c). Figure 5b shows the effect on the MR signal acquired with different TS during the same bolus injection. For shorter TS the MR signal perfectly follows the contrast bolus, whereas for longer TS the MR signal is fully relaxed, reaching a plateau that limits the ability of the MR signal to track the highest contrast concentrations. The R1 values obtained after fitting the saturation recovery experiment for each time domain point were normalized to those obtained before contrast injection (ΔR_1_) (Figure 5d).

**Figure 6:**
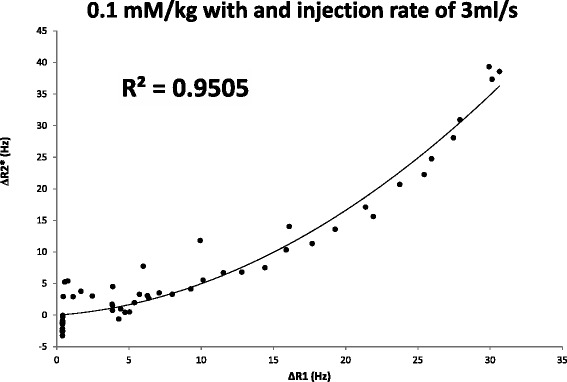
Correlation between R1 and R2* during same in-vivo bolus injection. Relation between the R2* variation and R1 variation obtained from Figure 5 for all dynamics (black dots) and the quadratic fitting between both quantities.

The relation between ΔR_2_* and ΔR_1_ (Figure 6) was fitted to a quadratic relation [23], yielding a correlation coefficient of R^2^ = 0.95 with a final relation between both quantities as follows:

$$ {\Delta \mathrm{R}}_2^{*} = 0.0332\ {\Delta \mathrm{R}}_1^2 + 0.165\ {\Delta \mathrm{R}}_1 - 0.0003 $$

The relation established in this equation between ΔR1 and ΔR2* effects was subsequently used for contrast estimation *in vivo*.

#### Correlation between the dual-bolus and dual-saturation methods

**Figure 7:**
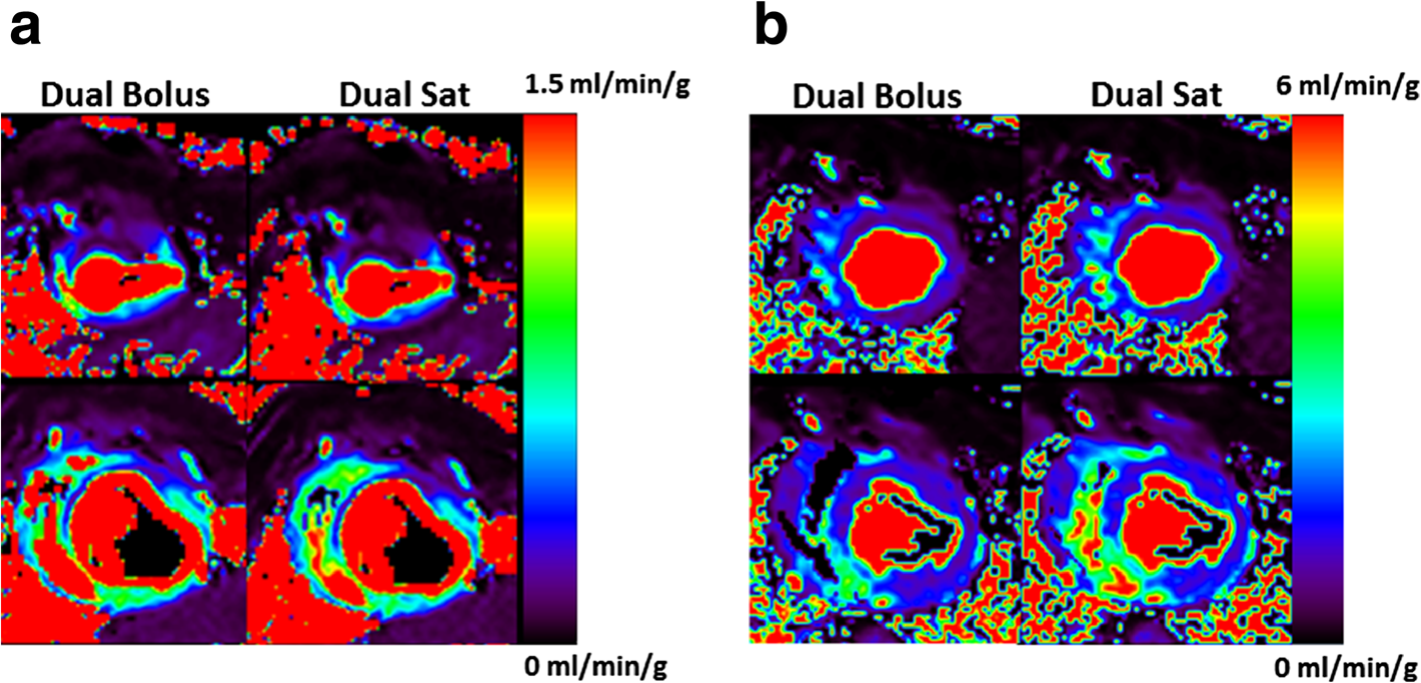
**Representative Myocardial Blood Flow maps.** Representative flow maps for left anterior descendant (LDA) coronary occlusion **(a)** and selected hyperemia at right coronary artery (RCA) **(b)** estimated with dual-bolus and dual-saturation strategies. In both cases dual-bolus and dual-saturation maps have equalized scales.

**Figure 8:**
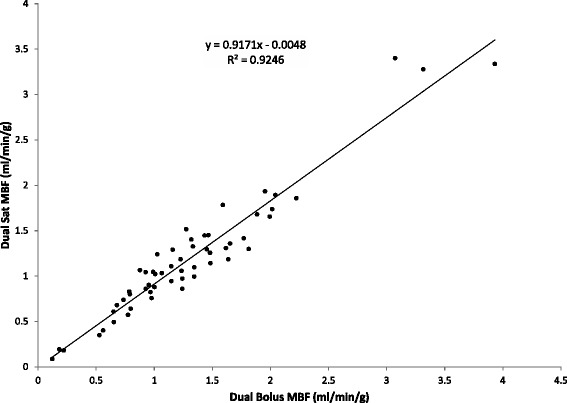
**Correlation analysis between Dual Bolus and Dual Saturation MBF.** Scatter plot and linear regression fitting (solid line) comparing the quantitative results obtained with the dual-bolus and dual-saturation approaches (black dots).

**Figure 9:**
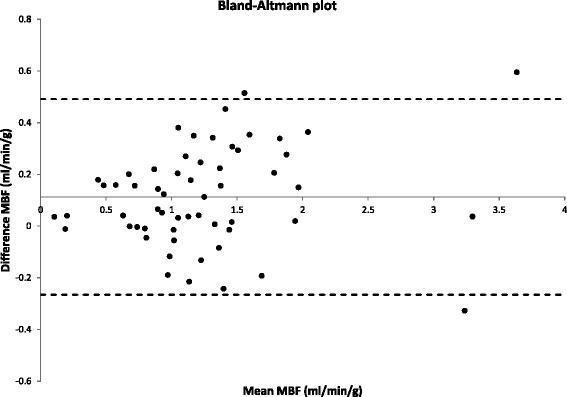
**Bias plot between Dual Bolus and Dual Saturation MBF.** Bland-Altman plot comparing the quantitative results obtained with the dual-bolus and dual-saturation approaches.

Representative MBF maps from both techniques are shown in Figure 7 for post infarction left anterior descending coronary artery (Figure 7a) and induced hyperemia of the right coronary artery (Figure 7b). Flow measurements obtained with the two methods (Figure 8) show a good linear relationship (R^2^ = 0.92). Bland-Altmann plot (Figure 9) shows a small disparity of 0.1 ml/min/g between the two perfusion measurements.

## Discussion

In this study we present a validation analysis for myocardial perfusion by dual-saturation acquisition including *in vitro* and *in vivo* experiments for the most important parameters affecting the quantification of absolute cardiac perfusion. The *in vitro* results were included in a comparison with the well-established dual-bolus technique in a pig model over a wide range of perfusion values. Our data show that a TS below 30 ms is sufficient to avoid AIF saturation. Although a low TE is desirable to avoid R2* effects on AIF estimation, we have also described the relation between R1 and R2* during bolus injection and included this in the conversion from signal to contrast concentration. The dual-saturation methodology was tested at flow values ranging from 0.12 to 3.92 ml/min/g, yielding a correlation coefficient with dual-bolus acquisition close to 1 (R^2^ = 0.92).

This correlation is slightly higher than that previously reported for analysis of the dual-bolus method with the multiple saturation recovery times (m-SRT) technique [24] (0.92 vs 0.82), probably due to the wider flow range explored in the present study. Moreover, overestimation of m-SRT quantitative perfusion has been reported at low perfusion rates [24]. In our study, measurements with the dual-bolus and dual-saturation methodologies show good agreement at low flow rates, with a constant level close to 0 (0.0048 ml/min/g). This discrepancy with the previous report can be explained by the use of higher contrast concentration in the present study. Direct comparison of contrast concentration between the two reports is difficult due to the use of different contrast agents (Gd-DPTA here and Gd-BOPTA in the previous report); however, here we use a fivefold higher contrast volume for *in-vivo* experiments (0.1 vs 0.02 mmol/kg), while the r1 relaxivity of Gd-BOPTA is just 1.31 times higher than that of Gd-DPTA [25].

Compared with the dual-bolus method, the dual-saturation approach underestimates flow at high flow rates, a result in agreement with previous findings [24]. This effect can probably be explained by physiological changes to the AIF in the dual-bolus approach at the high contrast concentration and under stress conditions, where it can be more difficult to maintain a constant and homogeneous vasodilation. In contrast, these changes are not seen when a single injection of contrast agent is used since AIF and cardiac muscle information are acquired at the same time.

The Bland-Altman plot showed that the mean difference between the two methods is 0.1 ml/min/g, which is the minimum flow detected by both techniques and is thus within the expected intrinsic error. The difference increases at higher myocardial flows, probably due to a higher heterogeneity of flow distribution in the cardiac muscle.

In order to obtain reliable perfusion measurements it is necessary to consider different potential factors that can influence estimation of contrast concentration changes in the blood pool (AIF) and cardiac muscle from the MR signal. One factor is a high contrast concentration passing through the ventricular cavity, which directly produces an underestimate of AIF due to over-long TS selection and R2* signal decay. A second factor is the readout effect of the cardiac muscle MR signal in high resolution images and its influence on the estimation of contrast concentration from the MR signal.

Accurate estimation of AIF first requires careful selection of the TS in order to avoid signal saturation at a too high contrast concentration. The results presented in Figure 3 show that in images acquired with TS values below or equal to 30 ms there is no substantial saturation effect on the MR signal for a maximum R1 of 71.69 Hz. Based on previously reported relaxivity values of Magnevist at 3 T [23], this value corresponds to a contrast concentration of 21.8 mM in aqueous solution (Γ = 3.29Hz/mM at 3 T) and 19.97 mM in blood (Γ = 3.59Hz/mM at 3 T). This is within the range of previous reported values for contrast concentration in the left ventricle cavity of healthy volunteers receiving an injection of Magnevist (0.1 mmol/kg) at a rate of 6 ml/s [16]. The figure also shows that a 200 ms TS allowed a more detailed analysis of contrast concentrations below 4.24 mM (R1 = 13.95Hz) using the full signal recovery range for this small contrast concentration window [6]. Unfortunately, at longer saturation times it is more difficult to acquire all slices in a single heartbeat, making it necessary to use shorter saturation times.

The second source of error that can affect AIF estimation at high contrast concentration is the potential for R2* effects. This effect can be observed in Figure 5a. During the same contrast injection different echoes are acquired with the corresponding R2* signal decay. With a contrast dose of 0.1 mmol/kg injected at a rate of 3 ml/s the maximum ΔR2* value was 39Hz, which underestimated AIF by 3.5% for the TE used in the present study (TE = 0.9 ms) and by 2.2% for previously reported TE (TE = 0.58 ms) [26]. This effect has been compensated for in the contrast conversion procedure by using the estimated relation between the R1 and R2* effects (Figure 4) in contrast conversion curves. To be able to estimate this relation, R1 and R2* were estimated simultaneously during the same contrast injection. Assuming a linear relation between contrast concentration and R1 changes [23], a quadratic relation was found between R1 and R2* values *in-vivo*, as previously reported [15,22].

Readout effects are more pronounced in high resolution images than in the AIF estimation due to the application of higher turbo factors before reaching the center of the k-space. This series of RF pulses influences magnetization and therefore the estimation of contrast concentration within the cardiac muscle. The proposed signal model demonstrates a good agreement with the acquired data, yielding a correlation coefficient close to 1.

### Limitations

The main limitation of the present study is that the dual-saturation method was compared to dual-bolus acquisition as a gold standard. Comparison with microspheres was not performed. However, the dual-bolus method has consistently shown good agreement in phantom analysis [27], with microspheres [10,28], and in nuclear medicine in human studies [4].

## Conclusions

The dual-saturation strategy for absolute quantification of myocardial perfusion by 3T-CMR shows an almost perfect correlation with the dual-bolus method across a wide range of perfusion scenarios (form post-infarction hypoperfusion to pharmacologically-induced hyperemic status). The dual-saturation strategy is moreover easier to implement in clinical protocols. Obtaining reliable flow measurements with the dual-saturation strategy requires careful selection of specific parameters. Chief among these are the correct selection of TS to ensure adequate evaluation of contrast uptake in the AIF (TS < 30 ms) and cardiac muscle (TS < 100 ms); the relation between the contrast injection protocol and the R2* effects on the AIF—always attempting to acquire images with the shortest possible TE; and the readout scheme for proper conversion of signal intensity changes into real contrast uptake.

## Appendix

For the contrast concentration used in clinical practice, a linear relationship can be assumed between the changes in contrast concentration and R1 variation in tissues. The first step to assess contrast concentration changes is the accurate evaluation of R1 changes (ΔR1) as the difference between the tissue R1_c_ after contrast injection and the baseline R1_b_ value. Accurate estimates of R1 variation changes are therefore required for a proper estimation of contrast concentration.

Conventional cardiac perfusion acquisitions are based on a saturation recovery experiment followed by different readout strategies normally based on acquisition techniques for single-shot spoiled turbo field echo (TFE) or hybrid spoiled TFE echo planar imaging (TFE-EPI). TFE acquisition is normally desired since shorter echo times are achievable, thus making this approach less sensitive to R2* [29]. The signal model described in this report can be applied to both TFE and TFE-EPI.

To obtain reliable R1 changes it is necessary to have a detailed understanding of the progress of magnetization between the saturation pulse and the center of the k-space (k0), where the contrast of the image is generated. In saturation recovery experiment (Figure 2) the MR signal recovers after the saturation pulse with the corresponding T1 value according to equation [A1]

A1 $$ {\mathrm{S}}_{\mathrm{T}0}={\mathrm{S}}_0{\mathrm{e}}^{-R{2}_b^{*}TE}\left(1-{e}^{-\mathrm{R}{1}_{\mathrm{b}}\mathrm{T}0}\right), $$

where T0 is the time between the saturation pulse and the starting point of the image readout; R1_b_ and S_0_ are R1 and the fully recovered signal intensity of the tissue; TE is the sequence echo time; and R2_b_* represents the transverse relaxation rate. After T0 the imaging readout sequence starts with different excitation pulses that affect the spin magnetization before reaching the center of the k-space, where the image contrast is defined. These signal changes can be expressed as:

A2 $$ {\mathrm{S}}_{\mathrm{b}}={\mathrm{e}}^{-R{2}_b^{*}TE}\left[{\mathrm{S}}_0^{*}-\left({\mathrm{S}}_0^{*}-{\mathrm{S}}_{\mathrm{T}0}\right){\mathrm{e}}^{-R{1}_b^{*}\left(\mathrm{T}\mathrm{S}-\mathrm{T}0\right)}\right], $$

where TS is the defined saturation time in the acquisition sequence, and $R{1}_b^{*}$ and ${S}_0^{*}$ are the modified R1 and steady-state magnetization of a spoiled gradient echo acquisition, which are related to original T1 and S_0_ values by:

A3 $$ \upepsilon =\frac{{\mathrm{S}}_0^{*}}{{\mathrm{S}}_0}=\frac{{\mathrm{R}}_1}{R_1^{*}}=\frac{\mathrm{T}\mathrm{R}}{\mathrm{T}\mathrm{R}-{\mathrm{T}}_1 \ln \left( \cos \left(\upalpha \right)\right)}, $$

where TR is the time between two consecutive excitation pulses and α is the TFE excitation angle. From equation [A2] and [A3] it can be shown that the signal will be more affected when more excitation pulses are applied between the saturation pulse and the acquisition of image k0. This difference can change with image resolution, profile order, half scan and parallel imaging acquisition factor.

The change in contrast concentration over time can be obtained from the ratio of signal intensity in the baseline situation before contrast injection (S_b_) to the signal intensity after contrast arrival (S_c_). By including the signal model described in Eq [A2] in the ratio it can be demonstrated that the signal relation follows the signal model described by

A4 $$ \frac{{\mathrm{S}}_{\mathrm{c}}}{{\mathrm{S}}_{\mathrm{b}}}{\mathrm{C}}_1={\mathrm{e}}^{-TE*\varDelta R{2}^{*}}\left[\upepsilon -\left(\upepsilon -\left(1-{\mathrm{e}}^{-R{1}_cT0}\right)\right){\mathrm{e}}^{-R{1}_c^{*}\left(TS-T0\right)}\right], $$

where

$$ {\mathrm{C}}_1=\upepsilon -\left(\upepsilon -\left(1-{\mathrm{e}}^{-R{1}_bT0}\right)\right){\mathrm{e}}^{-R{1}_b^{*}\left(\mathrm{T}\mathrm{S}-\mathrm{T}0\right)}\ \mathrm{and}\ \frac{1}{\mathrm{T}{1}_{\mathrm{c}}}=\mathrm{R}{1}_{\mathrm{c}}=\mathrm{R}{1}_b+\varDelta \mathrm{R}1 $$

Equation A4 states that with knowledge of the baseline T1 obtained by MOLLI sequence [21], the concentration of the contrast agent can be obtained at any time domain point by using the baseline signal as a normalization factor.
